# Th1 cytokines synergize to change gene expression and promote corticosteroid insensitivity in pediatric airway smooth muscle

**DOI:** 10.1186/s12931-022-02046-1

**Published:** 2022-05-16

**Authors:** Devine Jackson, Joshua Walum, Priyanka Banerjee, Brandon W. Lewis, Y. S. Prakash, Venkatachalem Sathish, Zhaohui Xu, Rodney D. Britt

**Affiliations:** 1 Centers for Perinatal Research, Abigail Wexner Research Institute at Nationwide Children’s Hospital, Columbus, OH 43215 USA; 2 Vaccines and Immunity, Abigail Wexner Research Institute at Nationwide Children’s Hospital, Columbus, OH USA; 3 Department of Pediatrics, The Ohio State University, Columbus, OH USA; 4 Department of Pharmaceutical Sciences, North Dakota State University, Fargo, ND USA; 5 Department of Anesthesiology and Perioperative Medicine, Mayo Clinic, Rochester, MN USA; 6 Department of Physiology and Biomedical Engineering, Mayo Clinic, Rochester, MN USA

**Keywords:** TNFα, IFNγ, Corticosteroids, Airway smooth muscle

## Abstract

**Background:**

Corticosteroids remain a key therapy for treating children with asthma. Patients with severe asthma are insensitive, resistant, or refractory to corticosteroids and have poorly controlled symptoms that involve airway inflammation, airflow obstruction, and frequent exacerbations. While the pathways that mediate corticosteroid insensitivity in asthma remain poorly defined, recent studies suggest that enhanced Th1 pathways, mediated by TNFα and IFNγ, may play a role. We previously reported that the combined effects of TNFα and IFNγ promote corticosteroid insensitivity in developing human airway smooth muscle (ASM).

**Methods:**

To further understand the effects of TNFα and IFNγ on corticosteroid sensitivity in the context of neonatal and pediatric asthma, we performed RNA sequencing (RNA-seq) on human pediatric ASM treated with fluticasone propionate (FP), TNFα, and/or IFNγ.

**Results:**

We found that TNFα had a greater effect on gene expression (~ 1000 differentially expressed genes) than IFNγ (~ 500 differentially expressed genes). Pathway and transcription factor analyses revealed enrichment of several pro-inflammatory responses and signaling pathways. Interestingly, treatment with TNFα and IFNγ augmented gene expression with more than 4000 differentially expressed genes. Effects of TNFα and IFNγ enhanced several pro-inflammatory genes and pathways related to ASM and its contributions to asthma pathogenesis, which persisted in the presence of corticosteroids. Co-expression analysis revealed several gene networks related to TNFα- and IFNγ-mediated signaling, pro-inflammatory mediator production, and smooth muscle contractility. Many of the co-expression network hubs were associated with genes that are insensitive to corticosteroids.

**Conclusions:**

Together, these novel studies show the combined effects of TNFα and IFNγ on pediatric ASM and implicate Th1-associated cytokines in promoting ASM inflammation and hypercontractility in severe asthma.

**Supplementary Information:**

The online version contains supplementary material available at 10.1186/s12931-022-02046-1.

## Background

Corticosteroid sensitivity is an important factor in determining asthma severity [1]. While mild to moderate asthma-related symptoms are managed effectively using inhaled corticosteroids (ICS) at low-moderate doses, children with severe asthma experience persistent airflow obstruction and more frequent exacerbations despite the use of higher dose ICS or oral corticosteroids [2]. Airway smooth muscle cells (ASM) are important structural cells that regulate airway function and secrete several cytokines and chemokines to contribute to airway inflammation [3]. Corticosteroids inhibit the enhancing effects of pro-inflammatory cytokines and growth factors on ASM hypercontractility, remodeling and inflammation [4]. The important contributions of ASM to airway inflammation, lung function, and exacerbations highlight the relevance of ASM corticosteroid sensitivity in asthma.

In recent years, there is an increasing appreciation for the contributions of non-Th2 inflammatory pathways in the pathogenesis of severe asthma. Multiple studies have reported increased levels of IFNγ, a Th1 cytokine, and Th1 lymphocyte infiltration in lung-derived samples from adults with severe asthma [5–7]. Wisniewski et al. identified the presence of Th1 airway inflammation in children with allergic and non-allergic severe asthma [8]. Flow cytometry analyses found CD3 + CD4 + IFNγ + T lymphocytes amongst cells collected from BAL, suggesting that Th1 inflammation promotes corticosteroid insensitivity in severe pediatric asthma. Studies have identified Th1 inflammation and increased TNFα levels in corticosteroid-insensitive children with obesity-related asthma [9, 10]. Additionally, mice with increased Th1 inflammation during allergic airway inflammation have increased corticosteroid insensitivity accompanied by persistent hyperresponsiveness and remodeling [11, 12]. These data highlight the influence of Th1 inflammation on corticosteroid insensitivity in asthma.

Th1 cytokines, TNFα and IFNγ, have substantial pro-inflammatory effects on ASM that contribute to airway inflammation, hyperresponsiveness, and remodeling in asthma [13], however little is known about their combined effects on ASM gene expression. TNFα and IFNγ are known to synergistically induce the production of several pro-inflammatory cytokines and chemokines, including CXCL10, a key T lymphocyte chemoattractant implicated in severe asthma [7, 14]. In addition to stimulating the production of cytokines and chemokines, TNFα is known to increase hypercontractility and proliferation [15, 16]. Although Th1 cytokines are implicated in severe asthma, the understanding of how corticosteroids modulate their effects on gene expression in ASM are unknown.

Recent studies show that exposure to both TNFα and IFNγ uniquely induces corticosteroid insensitivity in human fetal (18–22 weeks gestational age) and adult ASM [17, 18]. To advance the understanding of how TNFα and IFNγ interactions mediate corticosteroid insensitivity, we performed RNA-seq on pediatric human ASM. Our novel findings are consistent with recent findings showing a unique interaction between TNFα and IFNγ that enables pro-inflammatory gene expression to persist in ASM treated with corticosteroids. This is supported by pathway enrichment and co-expression analyses showing a unique effect of TNFα and IFNγ on gene expression. We identified several pro-inflammatory genes and pathways related to ASM and it’s contributions to asthma pathogenesis that persists in the presence of corticosteroids. Collectively, our studies show that combined exposure to TNFα and IFNγ impairs corticosteroid sensitivity in ASM and may contribute to persistent symptoms and exacerbations in severe pediatric asthma.

## Methods

### Cell treatment and experimental design

Human pediatric (ASM) cells were isolated from trachea and lung biospecimens collected by Comprehensive Transplant Center Human Tissue Biorepository at the OSU Wexner Medical Center and Mayo Clinic. Samples used in this study are from nonasthma individuals who were 0–22 years old. Biospecimens are from individuals who underwent tracheal reconstruction surgery or donor lungs that did not meet criteria for lung transplantation. These studies were approved by Nationwide Children’s Hospital and Mayo Clinic Institutional Review Boards. Pediatric ASM cells were cultured in DMEM/F12 medium supplemented with 10% fetal bovine serum and 1% antibiotic–antimycotic at 5% CO_2_ and 37 °C. Cells were serum-starved with 1% antibiotic–antimycotic DMEM/F12 for 24 h. Cells were then treated with vehicle (0.001% DMSO), 10 nM fluticasone propionate (FP), 0.1–10 µM JAK Inhibitor I, 10 ng/mL TNFα, and/or 25 ng/mL IFNγ for 3, 6, 12, 18, or 24 h. Cells lysates and media were harvested and stored at − 80 °C until molecular analyses. Cytokines were purchased from R&D Systems (Minneapolis, MN), fluticasone propionate and JAK Inhibitor I from Millipore-Sigma (St. Louis, MO).

### ELISA

Duoset ELISA kits (R&D Systems) were used to measure CCL5 and CXCL10 secretion levels following the manufacturer’s protocol [17]. Absorbance was read at 450 and 530 nm using the SpectroMax M2e Spectrophotometer (Molecular Devices, San Jose, CA). Standard curve was generated to calculate the chemokine concentration in media samples.

### RNA-sequencing

Following treatment for 18 h, cells were harvested, and total RNA was extracted from each treatment group with Trizol reagent, digested with RNase-free DNase I, and then stored at – 80 °C. Frozen samples were shipped to Ocean Ridge Biosciences (Deerfield Beach, Fl) who performed library preparation and RNA sequencing. In brief, the samples were re-purified using Agencourt RNClean XP beads (cat no. A63987; Beckman Coulter Life Sciences, Indianapolis, IN). RNA quality was verified by gel electrophoresis, and total RNA concentration was measured using a spectrophotometer. RNA integrity was visualized by agarose gel with each sample exhibiting intact RNA as indicated by strong 28S and 18S bands and minimal degradation. To generate cDNA libraries from total RNA, 200 ng RNA was amplified using the Universal Plus mRNA-Seq Library Prep Kit (cat no. 0508-96; NuGEN Technologies, San Carlos, CA). Library size and quality were verified using chip-based capillary electrophoresis on Agilent 2100 Bioanalyzer High Sensitivity DNA assays (cat no. 5067-4626; Agilent Technologies, Santa Clara, CA) and quantified via Takara Library Quantification Kit (cat no. 638324; Takara Bio, San Jose, CA). For sequencing, libraries were pooled and loaded onto an Illumina cBot flow cell. Following extension and amplification to create sequence clusters, flow cells were transferred to the HiSeq4000 instrument and sequenced using 150 bp paired-end reads plus a single index read. Samples that did not reach the targeted sequencing depth (40 million total reads) were sequenced again using the NextSeq500 instrument. The RNA-seq dataset was deposited in GEO (www.ncbi.nlm.nih.gov/geo) under accession number GSE179354.

### Quality control and read alignment

RNA-seq data were analyzed on the NCH Franklin Cluster within the Anaconda environment for Python [19] and R scripts. FASTQ files were analyzed for quality before and after trimming with FASTQC [20] and then for batch effects and other potential confounding influences, age and gender, using Deeptools v3.3.1 [21]. Adaptors and other sequencing artifacts were removed using the bbtools v38.86 and bbduk tool with the default adaptors list [22]. Filtered and base-trimmed reads were then aligned using Hisat2 v2.2.1 and the GRCh38.p13 reference human genome [23]. Technical replicates were merged at this point of the pipeline, and the resulting SAM files were converted to BAM files, then sorted and indexed using SAMtools v1.9 [24]. Strandedness was verified using RSeQC v3.0.0 9 to establish settings for counting genomic features [25]. Following alignment, features were counted using HTSeq2 v0.11.2 and count tables generated [26]. A custom Python script was used to merge counts files into a single table, parse for duplicated stable ENSEMBL IDs, and merge duplicates.

### Identification and annotation of differentially expressed genes

Counts were normalized and differential expression between treatment groups (Vehicle, FP, TNFα, IFNγ) was determined using EdgeR v3.32.0 [27, 28]. Prior to performing differential expression analysis, the data was adjusted for fixed and random effects, gender, and age using a linear model. In addition to pairwise comparisons between each treatment and control, pairwise comparisons between FP treated and non-FP treated samples were performed. The treatments were arranged as follows: (1) Control, (2) FP, (3) TNFα, (4) FP + TNFα, (5) IFNγ, (6) FP + IFNγ, (7) TNFα/IFNγ, (8) FP + TNFα/IFNγ. The resulting data was annotated with the Bioconductor Homo.sapiens package v1.3.1 [29, 30]. False Discovery Rate (FDR) was calculated from the p-values using the Benjamini–Hochberg method. Genes considered differentially expressed had a log_2_ fold change of > 1.5 (up-regulated) or < − 1.5 (down-regulated) and an FDR < 0.05. Data exploration and visualization were performed using RStudio inside the Bioconductor environment for Principal Component Analysis (PCAtools) and generation of volcano plots (EnhancedVolcano) [30].

### Pathway and transcription factor enrichment analysis

Pathway and transcription factor enrichment of the identified differentially expressed genes, compared to control, was assessed using the g:Prolifer tool [31] in BioConductor. Due to the large number of differentially expressed genes identified in TNFα/IFNγ treatment groups, a threshold of log_2_ fold change of > 2 or < − 2 was used to characterize and manipulate the gene list in g:Profiler analysis. KEGG and Reactome terms and transcription factor motifs with p-value < 0.05 were considered significant. Terms and transcription factor motifs were ranked by precision (intersection/query size) and the top 10 terms or motifs are highlighted in the figures (results section).

### Co-expression and functional annotation analysis

Co-expression analysis on differentially expressed genes was performed using CEMiTool in BioConductor [32]. Data was reported in a html output file that included results from gene set enrichment analysis, over representation analysis, and interaction networks. Quality control data was reported in the diagnostics html output which includes sample clustering, mean variance, and mean connectivity. Associations amongst correlated genes were assigned using a soft-threshold beta = 8 and determination coefficient (R^2^ = 0.84). Gene set enrichment analysis identified modules that are enhanced or repressed within each treatment group. Over-representation analyses were also performed and showed Reactome Pathway enrichment in each module. Mean expression within each module is presented in profile plots. Genes within selected networks within significant modules were analyzed for functional annotation analysis using DAVID v6.8 [33]. Gene ontology terms associated with annotation clusters within each network were determined by enrichment score (> 2) and p-value (< 0.05).

### Statistical analyses

An individual pediatric ASM samples (n = 3–5) was used for ELISA experiments. Data were analyzed using GraphPad Prism 8.2.0 (GraphPad, San Diego, CA). One-way or two-way (factors: time, treatment) ANOVA with Sidak test for multiple comparisons was performed as appropriate. Values are expressed as mean ± standard error (SE) and p-value < 0.05 was considered statistically significant.

## Results

### TNFα/IFNγ induces corticosteroid insensitivity

**Fig. 1:**
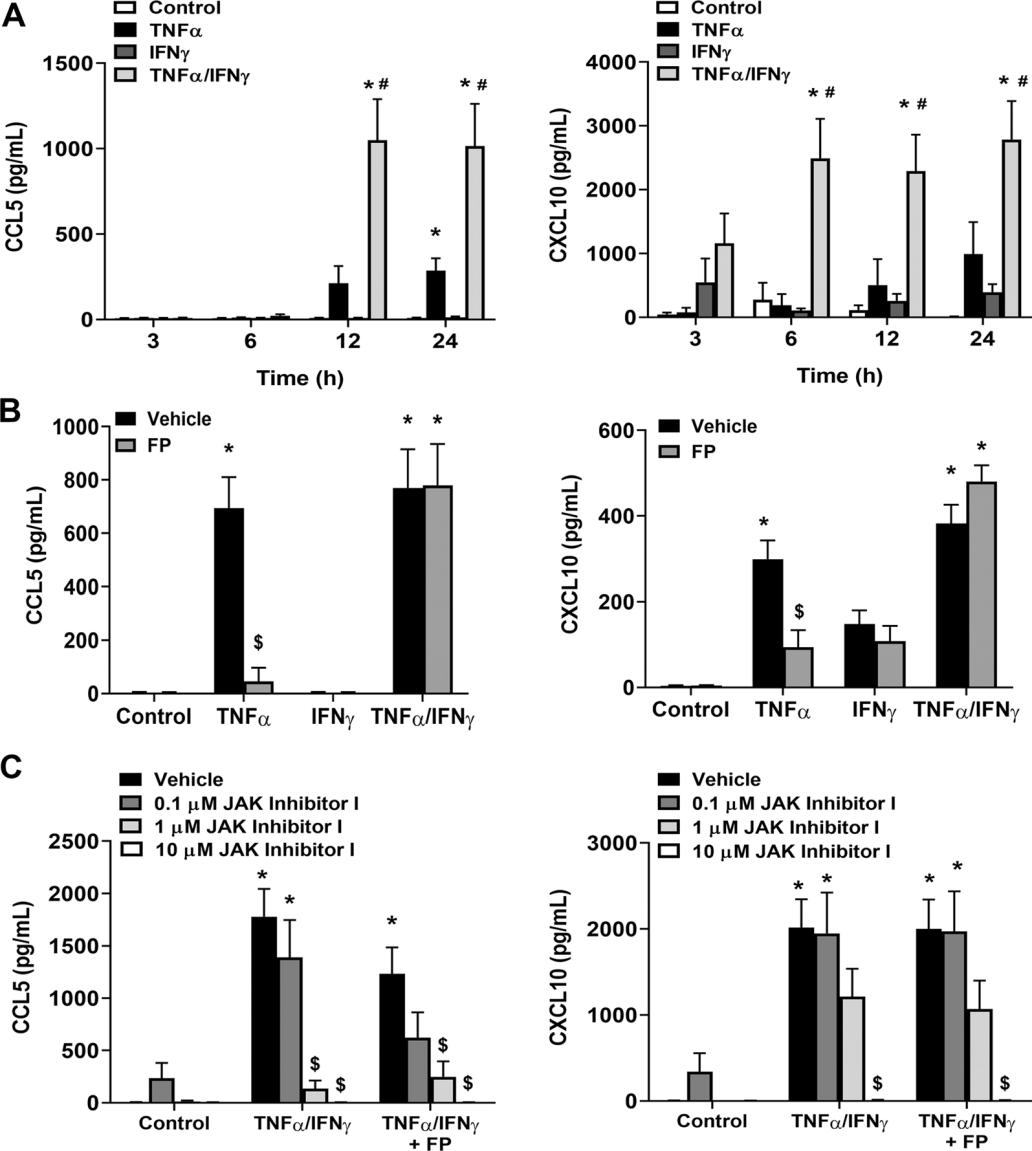
TNFα/IFNγ augments chemokine secretion that is insensitive to corticosteroids. **A** Cells were treated for 3, 6, 12, or 24 h. CCL5 and CXCL10 secretion are further increased by TNFα/IFNγ. **B** In contrast to TNFα or IFNγ alone, TNFα/IFNγ-induced CCL5 and CXCL10 secretion are insensitive to fluticasone propionate (FP) treatment. **C** JAK inhibition significantly reduces CCL5 and CXCL10 secretion. Data are presented as means ± SE, n = 3–5 individual ASM samples. *p < 0.05, significant difference from control; ^#^p < 0.05, significant difference from TNFα alone; ^$^p < 0.05, significant effect of FP or JAK inhibitor I

Human pediatric airway smooth muscle (ASM) cells were treated with TNFα, IFNγ, or TNFα/IFNγ from 3 to 24 h, and CCL5 and CXCL10 secretion levels were measured. TNFα alone increased CCL5 secretion after 24 h, while TNFα/IFNγ treatment significantly increased CCL5 expression in 12 h. Similarly, CXCL10 secretion was maximally increased by TNFα/IFNγ in 6 h (Fig. 1A). To assess corticosteroid sensitivity, cells were treated with 10 nM fluticasone propionate (FP), a concentration previously shown to be effective in developing ASM [17]. Treatment with FP significantly reduced CCL5 and CXCL10 secretion in ASM cells treated with TNFα; however, FP did not reduce the effects of TNFα/IFNγ (Fig. 1B). We treated ASM with vehicle, 0.1, 1, or 10 µM JAK inhibitor I, a reversible inhibitor of JAK protein-tyrosine kinase activity. Treatment with JAK inhibitor I inhibited TNFα/IFNγ-induced on CCL5 and CXCL10 secretion (Fig. 1C).

### Corticosteroid sensitivity and the individual effects of TNFα and IFNγ on gene expression

**Fig. 2:**
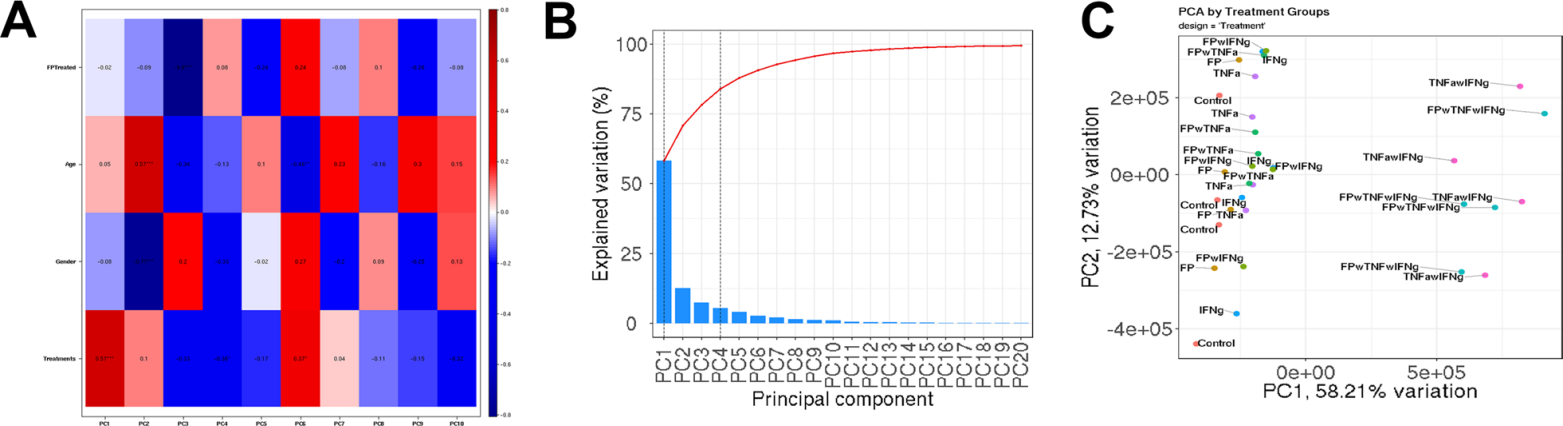
Principal Component Analysis of RNA-seq data. **A** The Eigencor plot shows effect of age, gender, fluticasone propionate (FP) and cytokine treatment on principal components. Significant effects were observed cytokine treatment (PC1), gender and age (PC2), and FP treatment (PC3). The scale bar represents Eigenvalues (− 1 to 1, with 0 having no correlation). adjusted p value < 0.05, ***indicates significant effect. **B** Percentage of variation for each principal component is shown in the Scree plot. **C** Principal Component Analysis plot for PC1 vs PC2, which together account for ~ 70% variation, is plotted

An RNA-Seq based approach was used to identify the genes differentially expressed in human pediatric ASM treated with vehicle, FP, TNFα, and/or IFNγ for 18 h. This time point was chosen based on previous RNA-seq studies involving corticosteroid responses in human ASM [34, 35]. On average, the sequencing generated 30.5 million reads among 48 samples and 95.2% reads were uniquely mapped to genes from the homo sapiens reference genome. After filtering, 17,434 genes from 8 groups were analyzed to identify differentially expressed genes. Principal component analysis identified significant treatment, gender, and age effects, as shown in the Eigencor plot (Fig. 2A). Further analyses show that treatment accounts for > 50% of changes in gene expression (Fig. 2B), with some contributions from age and gender. Plotting the first principal component (PC1) (treatment) vs. PC2 (gender) shows TNFα/IFNγ treated cells separate from all other treatment groups (Fig. 2C).

**Fig. 3:**
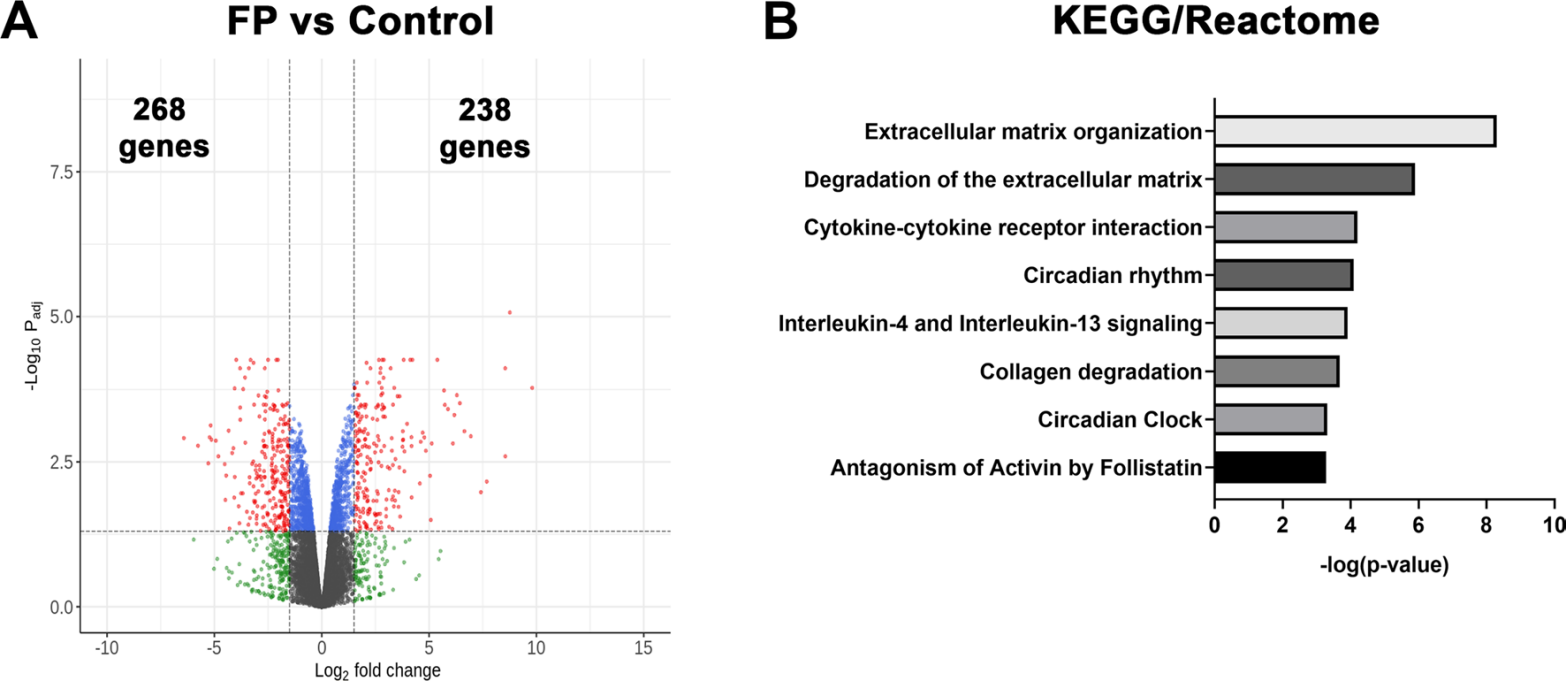
Differential gene expression and pathway analysis in response to fluticasone propionate (FP) alone. **A** Volcano plot for differentially expressed genes in FP vs. control. The total number of significantly up-regulated and down-regulated genes are stated. Volcano plots are represented by plotting -log_10_ (adjusted p value) and log_2_ fold change **B** Top 10 KEGG and Reactome terms. Enrichment is represented by plotting -log_10_ (adjusted p value)

Differentially expressed genes from each treatment group and their pairwise comparisons with other treatments are given in Additional file 1. The KEGG and Reactome pathways from g:Prolifer analysis in each treatment and comparison groups are provided in Additional file 2. Differential expression analysis shows that treatment with FP alone increased expression of 238 genes while 268 genes were down-regulated (Fig. 3A). Amongst the genes increased by FP, include those commonly associated (DUSP1, FKBP5, KLF15, PER1, TSC22D3) and not as commonly associated (ALDH1L1, ALOX15B, ZBTB16) with glucocorticoid receptor activity and anti-inflammatory mechanisms in ASM (Table 1). Interestingly, FP alone significantly reduced several pro-inflammatory genes related to ASM and asthma, including IL-6, MMP1, PTGS2, and TNFSF15 (Table 1). Enriched KEGG and Reactome terms include cytokine-cytokine receptor interaction, circadian rhythm, IL-4 and IL-13 signaling, and extracellular matrix organization (Fig. 3B). These data show that corticosteroids modulate anti- and pro-inflammatory gene expression in the absence of cytokine stimulation.

**Table 1:** Gene expression of notable corticosteroid sensitive genes

Gene symbol	Gene Name	FP		TNFα		FP + TNFα		IFNγ		FP + IFNγ		TNFα/IFNγ		FP + TNFα/IFNγ	
		Log_2_ Fold Change	FDR	Log_2_ Fold Change	FDR	Log_2_ Fold Change	FDR	Log_2_ Fold Change	FDR	Log_2_ Fold Change	FDR	Log_2_ Fold Change	FDR	Log_2_ Fold Change	FDR
ALDH1L1	aldehyde dehydrogenase 1 family member L1	8.76	0.0000	-1.53	0.6620	2.02	0.2446	2.35	0.3112	7.14	0.0000	− 3.74	0.2402	1.20	0.4478
ALOX15B	arachidonate 15-lipoxygenase type B	8.55	0.0000	1.89	0.4977	8.02	0.0016	3.82	0.1974	7.37	0.0001	7.31	0.0006	11.37	0.0000
FKBP5	FKBP prolyl isomerase 5	5.70	0.0002	0.004	0.9944	4.73	0.0014	2.52	0.0387	6.23	0.0001	2.12	0.0020	3.65	0.0000
IL6	interleukin 6	− 2.54	0.0042	6.39	0.0007	4.02	0.0062	1.84	0.0573	− 1.46	0.0314	4.67	0.0006	1.69	0.0752
KLF15	Kruppel like factor 15	3.76	0.0017	− 1.43	0.0165	2.17	0.045	− 0.40	0.5341	2.58	0.0014	− 3.96	0.0021	− 3.96	0.0021
MMP1	matrix metallopeptidase 1	− 4.06	0.0002	3.58	0.0185	− 2.46	0.0148	− 0.29	0.7233	− 2.87	0.0003	4.24	0.0000	− 0.63	0.3018
PER1	period circadian regulator 1	2.83	0.0002	0.76	0.0689	2.49	0.0075	0.25	0.3944	2.46	0.0006	0.8007	0.0308	2.65	0.0005
PTGS2	prostaglandin-endoperoxide synthase 2	− 2.73	0.0285	3.19	0.0025	− 1.50	0.2433	1.78	0.0004	-0.52	0.4137	5.22	0.0002	1.92	0.0044
TNFSF15	TNF superfamily member 15	− 2.90	0.0036	5.19	0.0149	1.39	0.290	0.25	0.8182	-2.20	0.1297	5.56	0.0008	2.3002	0.0327
ZBTB16	zinc finger and BTB domain containing 16	9.80	0.0002	− 0.68	0.6542	9.54	0.0032	2.29	0.5185	9.39	0.0000	0.04	0.9606	9.01	0.0002

**Fig. 4:**
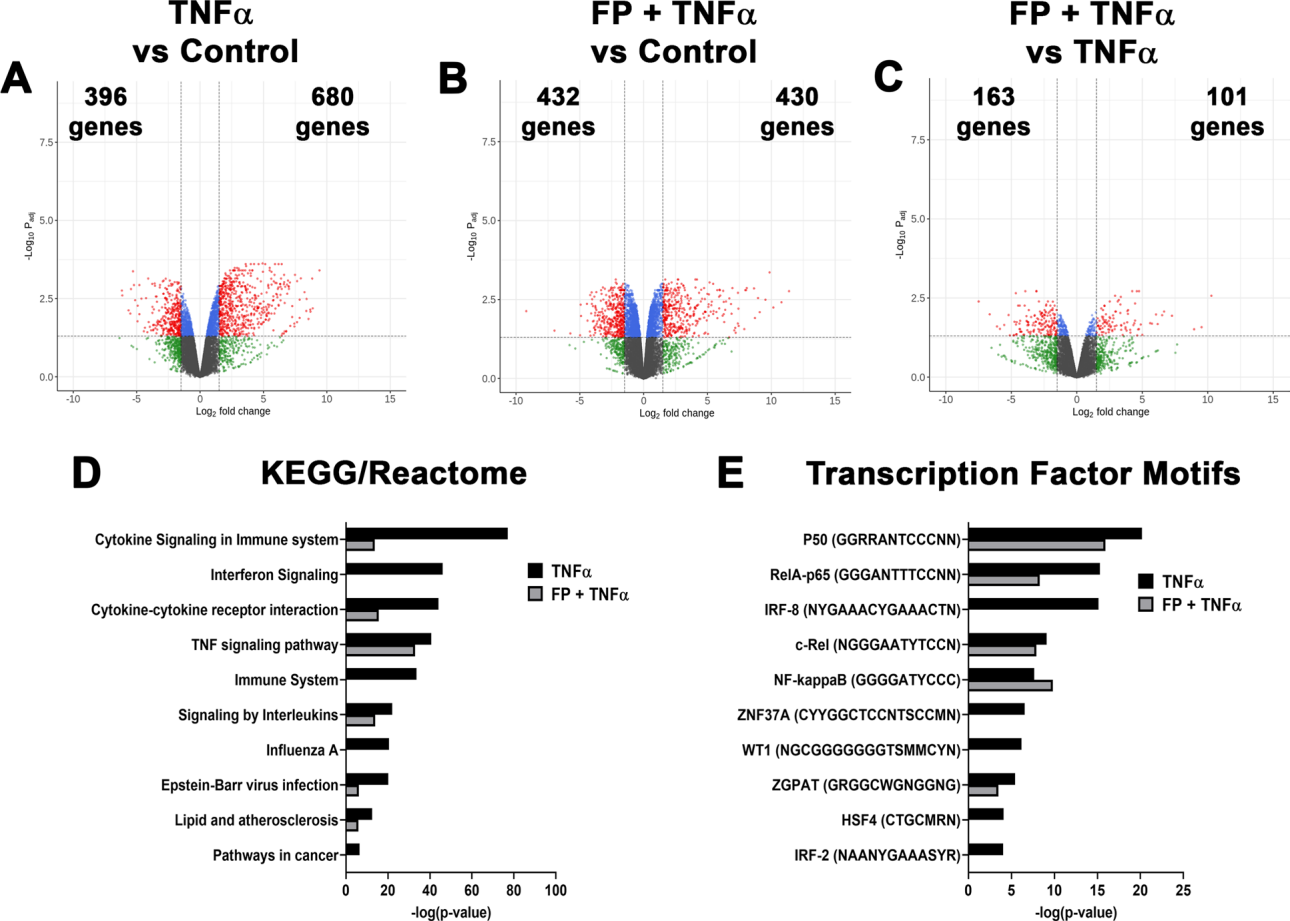
Differential gene expression and pathway analysis in response to TNFα and fluticasone propionate (FP). Volcano plots for differentially expressed genes in **A** TNFα vs. control, **B** FP + TNFα vs. control, and **C** FP + TNFα vs. TNFα are displayed. Volcano plots are represented by plotting -log_10_ (adjusted p value) and log_2_ fold change. The total number of significantly up-regulated and down-regulated genes are stated. Top 10 **D** KEGG and Reactome terms and **E** enriched transcription factor motifs. Enrichment is represented by plotting -log_10_ (adjusted p value)

Compared to controls, TNFα significantly regulated 1,076 genes, with 680 up-regulated and 396 down-regulated (Fig. 4A). Expression of several pro-inflammatory genes, where CCL5, CR1L, CXCL8, and TNFRSF9 were among the most increased by TNFα, while COL26A1, KRT4, PTGDR2 and Wnt11 were among the most reduced. Several KEGG and Reactome terms related to the immune system, TNFα and interferon signaling, and virus infection were significantly enriched pathways. We also found enriched transcription factor motifs (p50, IRFs, NFκB, RelA, c-Rel) associated with pro-inflammatory signaling induced by TNFα (Fig. 4D and E). Compared to TNFα alone, cells treated with FP + TNFα treatment significantly reduced expression of 163 genes induced by TNFα, including CCL5, IL6, MMP1, and PTGS2 (Fig. 4B and C; Table 1). We also observed that FP reduced enrichment in multiple pathways and transcription factors, including interferon signaling, immune system, influenza A, IRF2, and IRF8 (Fig. 4D and E).

**Fig. 5:**
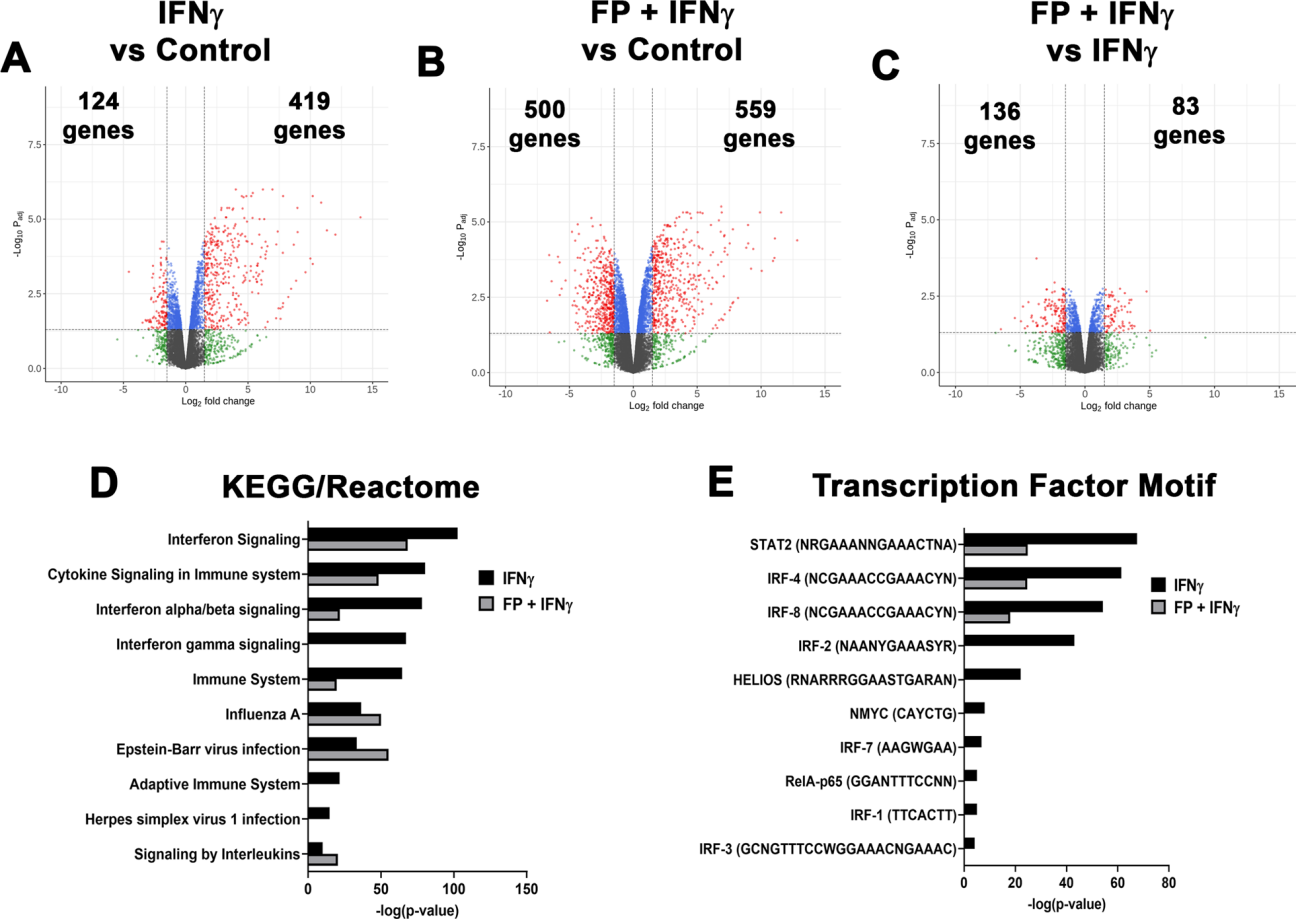
Differential gene expression and pathway analysis in response to IFNγ and fluticasone propionate (FP). Volcano plots for differentially expressed genes in **A** IFNγ vs. control, **B** FP + IFNγ vs. control, and **C** FP + IFNγ vs. IFNγ are displayed. The total number of significantly up-regulated and down-regulated genes are stated. Volcano plots are represented by plotting -log_10_ (adjusted p value) and log_2_ fold change. Top 10 **D** KEGG and Reactome terms and **E** enriched transcription factor motifs. Enrichment is represented by plotting -log_10_ (adjusted p value)

ASM exposed to IFNγ had 543 differentially expressed genes, with 419 up-regulated and 124 down-regulated (Fig. 5A). Among the genes most increased by IFNγ are those involved in antigen presentation (HLA-DRA, HLA-DOA), pro-inflammatory cytokines (CCL13, CXCL11, CX3CL1, CSF1), and adhesion molecules (ICAM1). Enriched KEGG and Reactome terms include genes involved in IFN signaling, viral infection, and adaptive immunity (Fig. 5D), while there were several enriched transcription factor motifs, including Stat2, IRFs, and RelA. Interestingly, most genes modulated by IFNγ were not significantly changed by FP (Fig. 5B and C). Among the pro-inflammatory genes that were significantly reduced were PTGS2, VCAM1, and TNFSF18 (Table 1). FP reduced enrichment of some KEGG and Reactome terms, IFNγ signaling and Adaptive Immune System, and several transcription factor motifs related to interferon signaling (IRF1, IRF2, IRF8, HELIOS) (Fig. 5D and E).

### Combined TNFα/IFNγ exposure augments gene expression in pediatric ASM

**Fig. 6:**
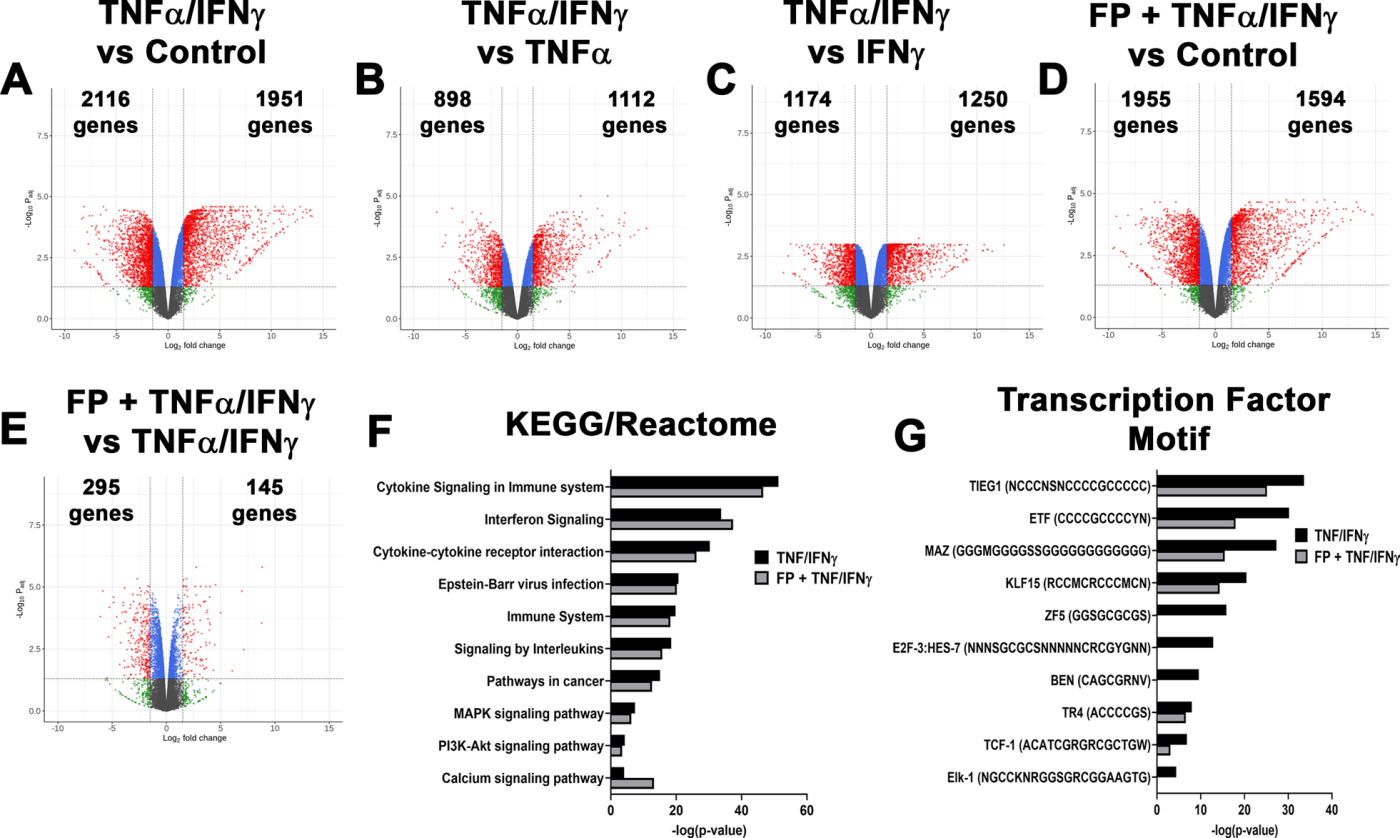
Differential gene expression and pathway analysis in response to TNFα/IFNγ and fluticasone propionate (FP). Volcano plot for differentially expressed genes in **A** TNFα/IFNγ vs. control, **B** TNFα/IFNγ vs. TNFα, **C** TNFα/IFNγ vs. IFNγ, **D** FP + TNFα/IFNγ vs. control, **E** FP + TNFα/IFNγ vs. TNFα/IFNγ are displayed. The total number of significantly up-regulated and down-regulated genes are stated. Volcano plots are represented by plotting -log_10_ (adjusted p value) and log_2_ fold change. Top 10 **F** KEGG and Reactome terms and **G** enriched transcription factor motifs. Enrichment is represented by plotting -log_10_ (adjusted p value)

Combined exposure to TNFα and IFNγ significantly increased the expression of 1951 and decreased 2116 genes, showing a unique expression profile in ASM treated with TNFα/IFNγ (Fig. 6A). Several pro-inflammatory mediators (CXCL9, CXCL10, CXCL11) and genes related to TNF- and IFN-signaling pathways (IRF1, IRF8, TRAF1) were significantly increased. We also noted an increase in the expression of Ca^2+^ regulatory proteins, including CD38 and Orai1. Notably, Orai1 was only increased by combined exposure to TNFα/IFNγ (Table 2). Among the genes significantly reduced by TNFα/IFNγ were CAMK2B, CYSLTR1, PDE1A, and WNT11. The differential analysis also revealed augmentation of gene expression in cells treated with TNFα and IFNγ compared to either cytokine alone (Fig. 6B and C). The expression of CCL5, CCL8, CXCL9, and IRF8 was augmented by TNFα/IFNγ (Table 2). We found 440 genes that remained sensitive to FP in TNFα/IFNγ treated cells (Fig. 6D and E), including genes increased by FP alone (PER1, ZBTB16). Conversely, we did observe that FP-induced KLF15 expression by FP was blunted by TNFα/IFNγ (Table 1). Pro-inflammatory genes that remained sensitive to FP during TNFα/IFNγ treatment were IL6, MMP1, PTGS2, and TNFSF15 (Table 1), while many augmented genes remained insensitive to FP (Table 2).

**Table 2:** Gene Expression of Notable Corticosteroid Insensitive Genes

Gene symbol	Gene Name	FP		TNFα		FP + TNFα		IFNγ		FP + IFNγ		TNFα/IFNγ		FP + TNFα/IFNγ	
		Log_2_ Fold Change	FDR	Log_2_ Fold Change	FDR	Log_2_ Fold Change	FDR	Log_2_ Fold Change	FDR	Log_2_ Fold Change	FDR	Log_2_ Fold Change	FDR	Log_2_ Fold Change	FDR
CCL5	C–C motif chemokine ligand 5	− 1.01	0.5441	9.41	0.0003	4.0	0.0056	2.25	0.0484	0.54	0.5766	13.41	0.0000	11.86	0.0001
CCL8	C–C motif chemokine ligand 8	− 3.04	0.3309	2.97	0.0495	1.24	0.4297	6.40	0.0002	5.16	0.0006	11.68	0.0000	10.04	0.0000
CD38	CD38 molecule	0.32	0.835	5.37	0.0013	3.93	0.0100	5.91	0.0000	5.09	0.0002	10.92	0.0001	10.95	0.0001
CXCL10	C-X-C motif chemokine ligand 10	− 0.14	0.9090	6.63	0.0005	2.95	0.0243	5.74	0.0004	4.23	0.0126	13.69	0.0000	13.5	0.0000
CXCL11	C-X-C motif chemokine ligand 11	− 0.23	0.8111	5.61	0.0007	2.23	0.1134	5.79	0.0003	6.84	0.0000	13.33	0.0000	13.351	0.0000
CXCL9	C-X-C motif chemokine ligand 9	0.03	0.9683	2.10	0.0292	1.34	0.3096	5.34	0.0028	5.65	0.0011	13.77	0.0000	5.65	0.0000
IRF8	interferon regulatory factor 8	− 0.08	0.9876	1.63	0.3988	3.85	0.0466	6.05	0.0011	6.18	0.0007	10.77	0.0000	10.16	0.0001
ISG15	ISG15 ubiquitin like modifier	− 0.99	0.2039	3.94	0.0020	0.84	0.4926	3.64	0.0003	3.70	0.0025	5.69	0.0003	5.36	0.0011
Orai1	ORAI calcium release-activated calcium modulator 1	− 0.13	0.6608	0.97	0.0660	0.61	0.0508	0.14	0.5912	-0.14	0.6397	2.47	0.0004	1.92	0.0008
VCAM1	vascular cell adhesion molecule 1	−3.56	0.0015	6.31	0.0011	3.38	0.0374	2.56	0.0000	0.95	0.0114	8.22	0.0003	7.55	0.0006

Although enrichment analysis was similar to the individual cytokine treatments, MAPK, PI3K-Akt, and Calcium signaling were additional KEGG and Reactome terms significantly enriched by TNFα/IFNγ (Fig. 6F). Conversely, the top transcription factor motifs enriched in TNFα/IFNγ treated ASM were different from individual cytokine treatments. Our analysis showed motif enrichment in TIEG1, ETF, MAZ, and KLF15 (Fig. 6G). At the same time, the motifs associated with TNFα- and IFNγ-mediated signaling were also significantly enriched (See Additional file 2: Profiler file). Most of the top enriched KEGG, and Reactome terms remained unaffected by FP treatment, while FP did reduce enrichment for ZF5, E2F, and BEN motifs (Fig. 6F and G).

### Differential co-expression and network interactions associated with TNFα and IFNγ signaling

**Fig. 7:**
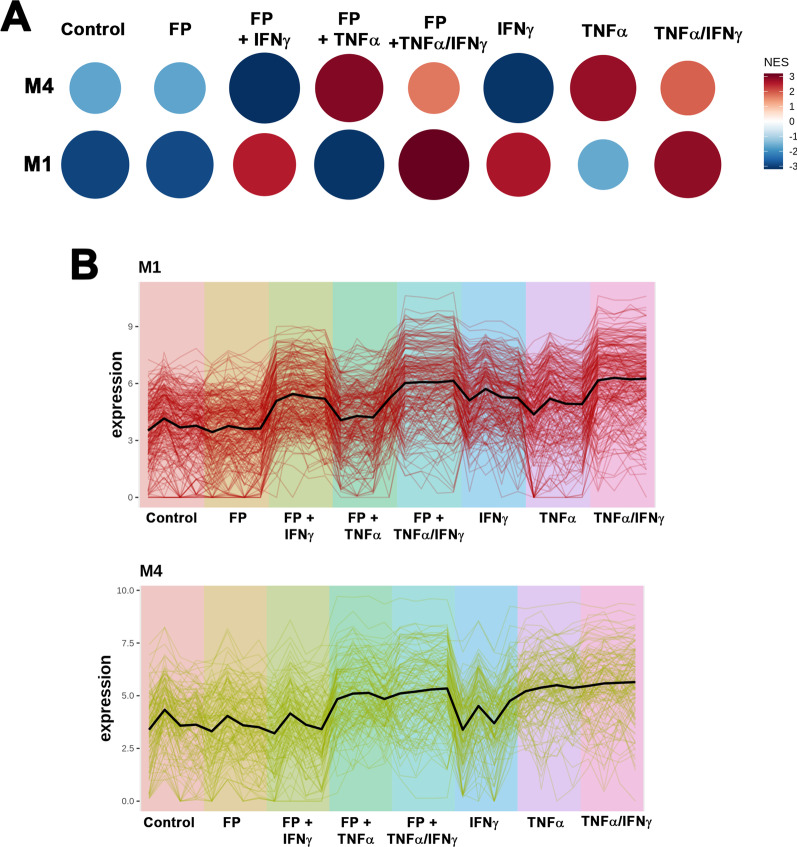
Co-expression analysis using CEMiTool. **A** Gene set enrichment analysis identified two modules, M1 and M4, that were found to be significant. Module activity for each treatment group is indicated by the dot size and color. The scale bar represents normalize enrichment score (NES). **B** Profile plots for M1 and M4. The black line represents mean gene expression within the module amongst each treatment group

**Fig. 8:**
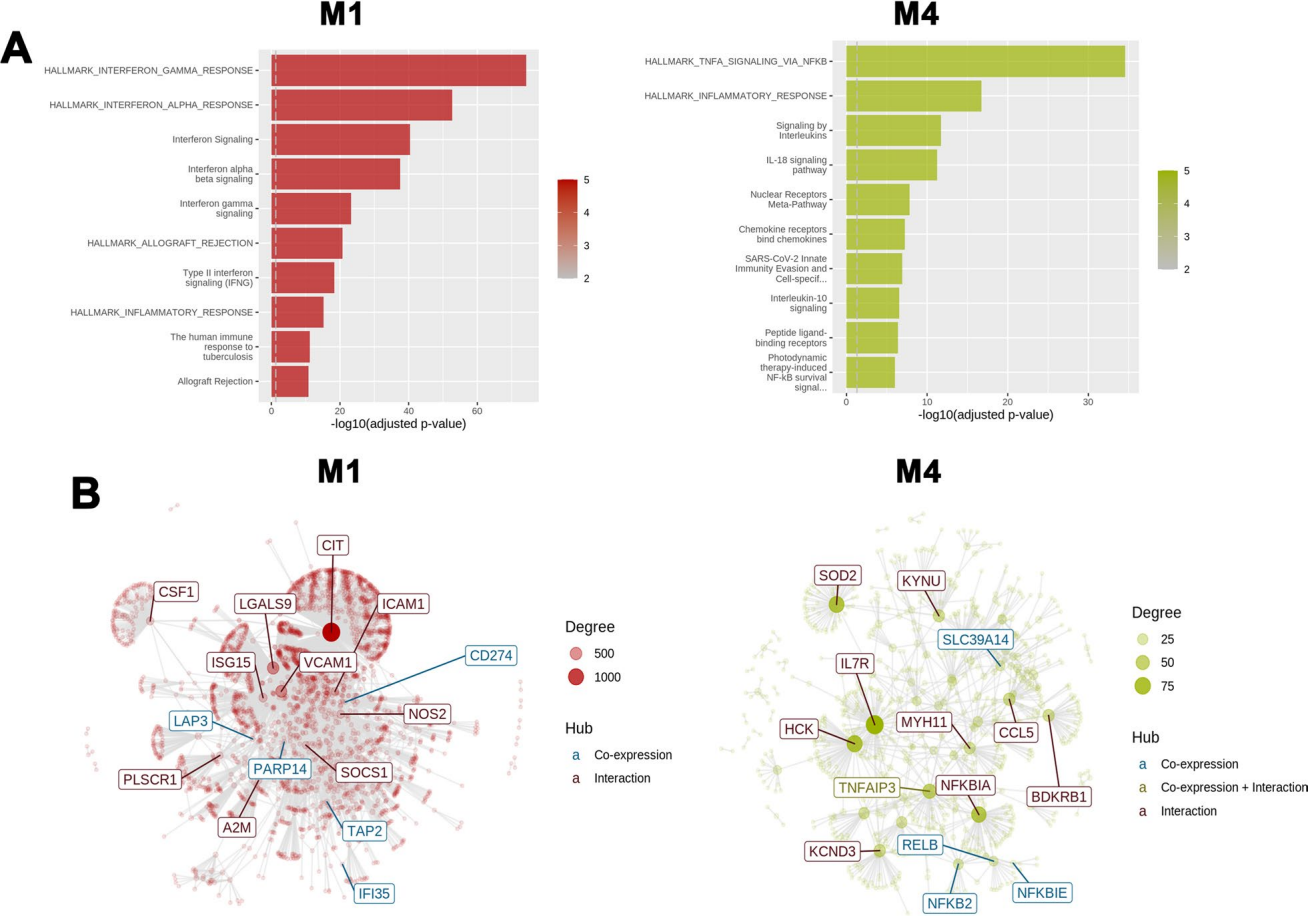
Gene enrichment analysis and interaction networks. **A** Over representation analysis identified pathways that are significantly enriched in modules 1 and 4. Notable pathways include IFN responses (M1) and TNFα and NFκB signaling in M4. Enrichment for each pathway is represented in the scale bar and plotted as -log_10_ (adjusted p value). **B** Gene networks for M1 and M4 are shown as connected gene hubs. Network connectivity and interactions are identified by node size and color intensity. Gene co-expression and interactions for each network hub are differentiated by color

To further understand biological relationships within our data, we performed co-expression analysis on our differential expression data set using CEMi Tool [32]. Gene set enrichment analysis identified five modules, however two modules, M1 and M4, had significant enrichment (p_*adj*_ < 0.05) and were biologically relevant with > 15 genes within enriched pathways (Fig. 7A). Profile plots for M1 and M4 show changes in mean gene expression (Fig. 7B). Gene networks and interactions for M1 and M4 show several networks or hubs with co-expression, interaction, or both. Results for network interactions and over-representation analysis are provided in Additional file 3. Over-representation analysis for M1 and M4 identified IFNγ and TNFα signaling pathways, respectively (Fig. 8A). Top gene hubs or interaction networks for M1 and M4 are shown in Fig. 8B. Notable interaction networks in M1 included genes associated with CIT, LGALS9, VCAM1, ISG15, and SOCS1. Networks interactions in M4 included genes associated had HCK, TNFAIP3, CCL5, BDKRB1, and MYH11 (Fig. 8B). The number of gene interactions, functional annotation analysis (DAVID functional annotation tool), and gene ontology terms within the top network interactions in M1 and M4 are given in Table 3, while the details of the interactions with the hub genes in M1 and M4 modules are shown in Additional file 4: Table S4 and Additional file 5: Table S5, respectively. Interaction networks in M1 and M4 are associated with cellular signaling pathways and functions, including RNA binding, cell–cell adhesion, JAK/Stat and NFκB signaling, and chemotaxis. Overall, co-expression analysis and functional annotation are consistent with differential and pathway enrichment analyses.

**Table 3:** Functional annotation for top interaction networks

Module	Co-expression Network Hub Gene	Number of Interactions	Number of Annotation Clusters	Top Functional Annotation Clusters within Co-expression Network
M1	CIT, Citron Rho-Interacting Serine/Threonine Kinase	1350	54	rRNA Processing, mRNA splicing, RNA binding, ATP binding, viral nucleoprotein
	LGALS9, Galectin	444	43	viral transcription, Cell–cell adhesion, nucleotide binding, protein folding, endoplasmic reticulum, mitochondrion, ER-Golgi transport
	VCAM1, Vascular Cell Adhesion Molecule 1	412	29	cell–cell adhesion, RNA-binding, protein folding, actin binding
	ISG15, ISG15 Ubiquitin-like Modifier	164	19	cadherin binding, ATP-binding, glycolytic process, actin binding, MHC class II protein complex binding
	SOCS1, Suppressor of Cytokine Signaling 1	86	24	ATP binding, MAPK cascade, tyrosine-protein kinase, JAK/Stat signaling pathway
M4	HCK, HCK Proto-Oncogene, Src Family Tyrosine Kinase	67	14	ATP binding, PI3K-Akt signaling, unfolded protein response, protein tyrosine kinase activity
	TNFAIP3, TNF Alpha Induced Protein 3	50	17	NFκB signaling, toll like receptor signaling, ubiquitin-protein transferase activity, CD40 receptor complex, TNF signaling
	BDKRB1, Bradykinin Receptor 1	30	2	Membrane, endoplasmic reticulum
	MYH11, Myosin Heavy Chain 11	19	5	cytoskeleton, motor protein, myosin, cell division
	CCL5, C–C Motif Chemokine Ligand 5	15	3	chemokine activity, leukocyte chemotaxis

## Discussion

Increased Th1 cytokines are associated with corticosteroid insensitivity and have been identified to be part of the inflammatory milieu in children with severe asthma [8, 9]. We have previously found that combined exposure to TNFα and IFNγ augments chemokine production and induces corticosteroid insensitivity in human fetal ASM [17]. Although glucocorticoid receptor signaling was not impaired in this model, we found evidence of continued NFκB and Stat1 activation in the presence of corticosteroids [17]. Similarly, we found that TNFα/IFNγ induces corticosteroid insensitivity in human pediatric ASM. While the effects of TNFα/IFNγ were insensitive to corticosteroids, JAK inhibition reduced the effects of TNFα/IFNγ, implicating JAK-mediated signaling in the combined effects of TNFα and IFNγ. However, it is unclear whether TNFα, IFNγ, or both contribute to JAK activation. The synergistic effects of TNFα and IFNγ have previously been shown to involve augmentation of CXCL10 that involve activation of JAK/Stat1, NFκB, and JNK signaling [14, 36, 37]. Building upon these studies, we performed RNA-seq to examine the individual and combined effects of TNFα and IFNγ on gene expression in the context of corticosteroid insensitivity in pediatric asthma. Our differential expression, pathway enrichment, and co-expression analyses provide novel insight into how corticosteroids modulate gene expression during exposure to TNFα, IFNγ, and TNFα/IFNγ.

In the absence of cytokines, transcriptomic analyses for ASM responses to corticosteroids have been previously reported in human ASM isolated from non-asthmatic and asthmatic lungs [34, 35, 38]. We observed similar changes in gene expression in ASM treated with FP alone, with comparable changes in gene expression. Notably, key anti-inflammatory genes demonstrated to inhibit inflammation and remodeling in human ASM [34, 39], DUSP1, TSC22D3, CRISPLD2 and KLF15, were substantially increased by FP. We also found that corticosteroids reduced several pro-inflammatory genes implicated in ASM inflammation and remodeling, including MMP1 and PTGS2 [40, 41]. These findings show that pediatric human ASM responds to corticosteroids, in the absence of cytokine stimulation, similarly to adult ASM.

TNFα has a potent effect on human ASM by inducing the production of pro-inflammatory mediators, extracellular matrix deposition and remodeling, and enhancing intracellular Ca^2+^ ([Ca^2+^]_i_) regulatory mechanisms that promote pathological and functional features in asthma [3, 13]. We found that TNFα changed expression of > 1000 genes, most of which were upregulated. Responses to TNFα involved increases in pro-inflammatory cytokines (IL-6, TNF, IL-1β), chemokines (CCL5, CXCL8), cell adhesion (ICAM, ICAM2), matrix metalloproteinases (MMP-9, MMP-13), and genes involved in TNF receptor signaling (TNFRSF9, TRAF1). We also noted several genes increased by TNFα that are involved in ASM [Ca^2+^]_i_ regulation and contractility (MYH11, CD38, BDKRB1, ANO9, SLC8A3). Exposure to corticosteroids changed the effects of TNFα on gene expression by significantly reducing more than 160 genes. Several pro-inflammatory genes increased by TNFα, including MMPs, CCL5, and PTGS2 were substantially reduced by corticosteroids. In pediatric ASM, TNFα-induced CCL5 secretion is blunted by FP, supporting observed changes in gene expression. These findings are consistent with studies showing potent anti-inflammatory effects of corticosteroids on human ASM treated with TNFα [42].

IFNγ also induces pro-inflammatory responses in ASM and has been implicated in mediating airway hyperresponsiveness in asthma [6, 12]. Among differentially expressed genes in response to IFNγ, the gene expression of pro-inflammatory mediators (CCL2, CCL8, CX3CL1), cell–cell adhesion molecules (ICAM1, CD40, VCAM1), and antigen presentation (HLA, PSME) were increased. Many of the pro-inflammatory genes enhanced by IFNγ remained increased in the presence of corticosteroids, suggesting differences in corticosteroid sensitivity between TNFα and IFNγ stimulation. For example, we found that CXCL10 gene expression and secretion induced by TNFα, but not IFNγ, was significantly reduced by FP. The corticosteroid insensitive effects of IFNγ may be attributed to reduced ability to reduce key JAK/Stat and IRF signaling pathways (IRF1, IRF8, JAK2, STAT1), which are enriched and are resistant to corticosteroids in human ASM [17, 43].

Treatment with TNFα/IFNγ induced a gene expression profile that is distinct from exposure to TNFα or IFNγ alone. TNFα/IFNγ augmented the expression of several genes that were also corticosteroid insensitive including, CD38, a Ca^2+^ regulatory protein that contributes to ASM hypercontractility and airway hyperresponsiveness [44, 45]. Additional notable genes that were augmented include CXCL11, IRF8, and VCAM1. Pathways related to genes modulated by TNFα/IFNγ involved pro-inflammatory signaling, infection, MAPK, and Ca^2+^ signaling. In addition to NFκB, IRF, and STAT pathways, we identified motif enrichment for several zinc finger transcription factors involved in DNA binding and gene regulation (ZF5, MAZ, TIEG1, KLF15). Zinc fingers are a large, diverse protein family with various cellular functions, including transcription factors that regulate gene expression. KLF15 is part of the kuppel like factor family and directly regulated by corticosteroids. KLF15 is involved in corticosteroid inhibition of TGFβ-induced ASM proliferation and remodeling [39, 46]. While KLF15 was increased by FP alone, it’s expression remained uniquely reduced in cells exposed to TNFα/IFNγ. While the implications of these findings remain unclear, we speculate that loss of KLF15 may contribute to corticosteroid insensitivity and enhanced gene expression upon exposure to TNFα/IFNγ.

To identify gene interactions and networks within our RNA-seq data, we performed co-expression analysis using CEMiTool [32]. Our analysis identified two modules that consist of interaction networks based on expression and biological function. Over-representation analysis revealed that networks in module 1 (M1) involved pathways related to IFNγ responses, while module 4 (M4) involved TNFα and NFκB signaling. The importance of TNFα- and IFNγ-mediated inflammatory pathways are highlighted by studies showing reduced inflammatory mediator production and [Ca^2+^]_i_ responses in ASM treated with siRNA or neutralizing antibodies against TNF receptors, NFκB, Stat1, and IRF1 [17, 43, 47]. Interaction networks in module 1 include gene hubs for IFN signaling (ISG15, SOCS1) and cell–cell adhesion (VCAM1, ICAM1, LGALS9). Module 4 had interaction networks that include chemokines (CCL5), NFκB signaling (TNFAIP3, RELB, NFKB2, and NFKBIE), and ASM contractility (MYH11 and BDKRB1). We performed functional annotation analysis to characterize the genes within the top co-expression networks. Highly enriched co-expression networks were composed of the genes involved in transcription, pro-inflammatory signaling, leukocyte chemotaxis, and smooth muscle contractility. Overall, co-expression and functional annotation analyses are consistent with g:Profiler pathway enrichment analyses and highlight the impact of TNFα and IFNγ on airway hyperresponsiveness and remodeling.

In addition to pro-inflammatory cytokines/chemokines and signaling, co-expression analysis in module 1 identified genes involved in cell–cell adhesion and antigen presentation. VCAM1 is an important adhesion molecule that facilitates ASM-T cell interactions and co-localization. It’s expression in ASM was found increased in bronchial biopsies from patients with asthma and is thought to promote ASM proliferation and remodeling [48]. Genes co-expressed with ISG15 were also found to be involved in MHC class II presentation. Co-culture ASM-T cell studies show induction of MHC class II expression in ASM and antigen presentation to resting T cells in vitro [49, 50]. Although ASM are likely not a primary antigen presentation cell in asthma, their increased expression of adhesion molecules and antigen presentation gene in response to TNFα and IFNγ highlight that ASM-T cell interactions may be important and warrant further investigation. Furthermore, the inability of corticosteroids to reduce TNFα/IFNγ-induced VCAM1 and antigen presenting genes implicate ASM-T cell interactions in persistent airway inflammation and thickening in severe asthma.

Co-expression analyses in module 4 revealed networks involved in ASM hypercontractility. TNFα and IFNγ increased myosin heavy chain 11 (MYH11) expression, a contractile protein that affects ASM shortening velocity. Studies show that MYH11 expression is increased in bronchial biopsies from patients with asthma, particularly of splice variants whose function increase shortening velocity and associated with greater airway narrowing [51–53]. While the effects of TNFα and IFNγ on myosin heavy chain 11 splice variant expression in human ASM are not entirely clear, these studies implicate their effects on MYH11 in promoting hypercontractility and bronchoconstriction. Additionally, our analysis identified bradykinin B1 receptor (BDKRB1) as a key network hub. BDKRB1 binds bradykinin, a pro-inflammatory mediator that acutely enhances [Ca^2+^]_i_ to promote hypercontractility and bronchoconstriction [54]. TNFα and IL-1β have been previously shown to increase expression of bradykinin receptors in mouse tracheal smooth muscle [55]. Recent studies have reported increased BDKRB1 expression correlates with airway remodeling and fixed airflow obstruction in severe asthmatic airways [56]. Additionally, exposure to TNFα enhances [Ca^2+^]_i_ responses in human ASM [57]. Our study suggests that MYH11 and BDKRB1 are enhanced during Th1 airway inflammation and highlight potential mechanisms by which TNFα and IFNγ promote ASM hypercontractility and remodeling.

Our transcriptomic analysis provides novel insight into gene networks and pathways associated with TNFα, IFNγ, and their corticosteroid sensitivity in ASM, however, there are limitations to consider. Corticosteroid sensitivity of pathways and networks are based on gene and not protein expression. This is important when considering the anti-inflammatory activity of genes enhanced by corticosteroids (e.g., DUSP1, KLF15) and highlights the need to examine corticosteroid sensitivity of pro-inflammatory genes and their activity at the protein level. The effects of corticosteroids are thought to involve several highly dynamic mechanisms. It is important to note that our analysis is only a snapshot into complex, and likely dynamic, changes in gene expression. Recent studies show that corticosteroids induce their initial anti-inflammatory effects on gene expression rapidly with 10–30 min [58]. Additionally, we found that initial inhibition of TNFα/IFNγ-induced CCL5 and CXCL8 mRNA expression by corticosteroid after 3 h was absent at later time points [17]. This suggests that timing is an important factor to consider regarding corticosteroid sensitivity and anti-inflammatory activity. Finally, our study does not inform on the impact of combined exposure to TNFα and IFNγ on ASM contractility. Future functional studies are needed to understand the effects of TNFα/IFNγ on human ASM contractility, particularly in response to bradykinin.

## Conclusions

We have identified several gene networks related to ASM responses to Th1 cytokines. Our analysis provides valuable insight into the individual and combined effects of TNFα and IFNγ of gene expression in pediatric ASM. TNFα/IFNγ was found to augment the expression of several genes that contribute to airway inflammation, hypercontractility and remodeling in asthma while also reducing expression of key anti-inflammatory genes, such as KLF15. It will be important to understand how genes augmented by TNFα/IFNγ are able to persist while glucocorticoid receptor activity is maintained. Additional factors relevant to corticosteroid sensitivity, such as chromatin structure, mRNA stability, and enzymatic activity of key negative regulators can be explored in future studies using the TNFα/IFNγ model of corticosteroid insensitivity [17, 58, 59]. Defining corticosteroid-sensitive and -insensitive genes/pathways in ASM (and other cell types) will be important for identifying novel approaches to enhance corticosteroid sensitivity in pediatric asthma.

## Supplementary Information

**Additional file 1.** Differential Gene Expression in pediatric ASM treated with control, FP, TNFα, IFNγ, and/or TNFα/IFNγ.

**Additional file 2.** KEGG/Reactome pathway and transcription factor enrichment results from g:Profiler.

**Additional file 3.** Interactions and over representation analysis results from CEMi Tool.

**Additional file 4.** Functional annotation results for notable Module 1 networks using DAVID.

**Additional file 5.** Functional annotation results for notable Module 4 networks using DAVID.

## Data Availability

Data generated and analyzed for this study has been deposited to the Gene Expression Omnibus (www.ncbi.nlm.nih.gov/geo) under accession number GSE179354.

## Abbreviations

ASM: Airway smooth muscle
FP: Fluticasone propionate
ICS: Inhaled corticosteroids
IFNγ: Interferon-γ
KEGG: Kyoto encyclopedia of genes and genomes
M: Module
PC: Principal component
RNA-seq: RNA sequencing
Th: T helper
TNFα: Tumor necrosis factor-α
